# Local hyperhemia to heating is impaired in secondary Raynaud's phenomenon

**DOI:** 10.1186/ar1785

**Published:** 2005-07-19

**Authors:** Aude Boignard, Muriel Salvat-Melis, Patrick H Carpentier, Christopher T Minson, Laurent Grange, Catherine Duc, Françoise Sarrot-Reynauld, Jean-Luc Cracowski

**Affiliations:** 1 Laboratory HP2, EA 3745 Inserm ESPRI, Grenoble Medical School, France; 2 Inserm Clinical Research Center 03, Grenoble University Hospital, Grenoble, France; 3 Vascular Medicine Department, Grenoble University Hospital, Grenoble, France; 4 Department of Human Physiology, University of Oregon, Eugene, Oregon, USA; 5 Department of Rheumatology, Grenoble University Hospital, Grenoble, France; 6 Internal Medicine Department, Grenoble University Hospital, Grenoble, France

## Abstract

Accurate and sensitive measurement techniques are a key issue in the quantification of the microvascular and endothelial dysfunction in systemic sclerosis (SSc). Thermal hyperhemia comprises two separate mechanisms: an initial peak that is axon reflex mediated; and a sustained plateau phase that is nitric oxide dependent. The main objective of our study was to test whether thermal hyperhemia in patients with SSc differed from that in patients with primary Raynaud's phenomenon (RP) and healthy controls. In a first study, we enrolled 20 patients suffering from SSc, 20 patients with primary RP and 20 healthy volunteers. All subjects were in a fasting state. Post-occlusive hyperhemia, 0.4 mg sublingual nitroglycerin challenge and thermal hyperhemia were performed using laser Doppler flowmetry on the distal pad of the third left finger. In a second study, thermal hyperhemia was performed in 10 patients with rheumatoid arthritis and 10 patients with primary RP. The thermal hyperhemia was dramatically altered in terms of amplitude and kinetics in patients with SSc. Whereas 19 healthy volunteers and 18 patients with primary RP exhibited the classic response, including an initial peak within the first 10 minutes followed by a nadir and a second peak, this occurred only in four of the SSc patients (p < 0.0001). The 10 minutes thermal peak was 43.4 (23.2 to 63), 42.6 (31 to 80.7) and 27 (14.7 to 51.4) mV/mm Hg in the healthy volunteers, primary RP and SSc groups, respectively (p = 0.01), while the 44°C thermal peak was 43.1 (21.3 to 62.1), 42.6 (31.6 to 74.3) and 25.4 (15 to 52.4) mV/mm Hg, respectively (p = 0.01). Thermal hyperhemia was more sensitive and specific than post-occlusive hyperhemia for differentiating SSc from primary RP. In patients with rheumatoid arthritis, thermal hyperhemia was also altered in terms of amplitude. Thermal hyperhemia is dramatically altered in patients with secondary RP in comparison with subjects with primary RP. Further studies are required to determine the mechanisms of this altered response, and whether it may provide additional information in a clinical setting.

## Introduction

Vascular dysfunction is a key element of the systemic sclerosis (SSc) disease process, and involves both the micro and macrovasculature [1]. The microcirculation undergoes structural and functional changes that are interdependent. This microangiopathy is characterized by capillary rarefaction, development of megacapillaries and vascular obliteration [2], which are associated with functional abnormalities mainly related to an endothelial dysfunction. Endothelial cells seem to play a pivotal role in SSc pathogenesis via the impairment of endothelium-dependent vasodilation and an increased transendothelial migration of T lymphocytes [3,4]. Endothelium-dependent vasodilation is impaired in patients with SSc mainly through an impaired ability to release nitric oxide (NO), and is an early event in the disease process [1,5]. Furthermore, patients with SSc have fewer endothelial progenitor cells than controls, and those present are often dysfunctional as well [6].

Accurate and sensitive measurement techniques are a key issue in the quantification of this vascular dysfunction, especially endothelial dysfunction. Different techniques have been used to quantify the microvascular dysfunction in SSc, such as microinjection [7], video microscopy [8], iontophoresis [9] or venous occlusion plethysmography [3], whereas endothelial function of conductance arteries can be monitored using ultrasonography of the brachial artery [10]. An easier non-invasive technique for monitoring cutaneous vascular function is the response to a given physiological challenge using cutaneous laser Doppler flowmetry. Using cold tests, the response of skin cutaneous blood flow does not significantly differ between primary Raynaud's phenomenon (RP) and SSc [11-13]. The response to brachial artery occlusion, however, gives more interesting results. Indeed, several authors showed a dramatic alteration of the amplitude and kinetics of post-occlusive hyperhemia in patients with SSc in comparison with primary RP or healthy controls [14,15], whereas an altered amplitude but not altered kinetics was described by Rajagopolan *et al*. [12]. Although the reproducibility of the method is debated, post-occlusive hyperhemia has been proposed for use as a tool to assess microvascular function during therapy in diseases such as atherosclerosis [16,17]. This post-occlusive hyperhemia is due both to metabolic and endothelium derived factors. We found, however, using microdialysis and laser Doppler flowmetry, that NO release is not directly involved in such as response [18], which limits the interest of post-occlusive hyperhemia as a test of endothelial function in SSc. In contrast, local hyperhemia to local heating in a small area of skin provides interesting information as thermal hyperhemia comprises two separate mechanisms: an initial peak that is axon reflex mediated; and a sustained plateau phase that is NO dependent [19,20]. Thermal hyperhemia might, therefore, be a better tool to assess both endothelial and microvascular function than post-occlusive hyperemia, and as such was recently investigated as a clinical tool to assess endothelial function in diseases such as chronic renal failure [21].

The main objective of our study was to test whether thermal hyperhemia in patients with SSc differed from that in patients with primary RP and healthy controls. The secondary objectives were: to compare the kinetics and amplitude of thermal hyperhemia in patients with local or diffuse SSc; to assess any relationship with the Rodnan skin score; and to determine the sensitivity and specificity of thermal hyperhemia in comparison to post occlusive hyperhemia in order to distinguish patients with SSc from those with RP. Given the altered response to local heating we observed in SSc, we also tested in a second study whether this was specific or not, enrolling patients with rheumatoid arthritis, another connective tissue disease that may present with RP.

## Materials and methods

### Patients

#### First study

The first study compared thermal hyperhemia in patients with systemic sclerosis with that in patients with primary Raynaud's phenomenon and healthy controls. We studied 60 consecutive subjects at the Inserm Clinical Research Center (Grenoble University Hospital, Grenoble, France) from January 2004 to October 2004: 20 patients suffering from systemic sclerosis, 20 patients with primary Raynaud's phenomenon and 20 healthy volunteers. These subjects are involved in a larger cohort study of the vascular phenotype of SSc. The criteria for inclusion in the study in the SSc cohort were diagnosis of SSc according to the criteria of the American College of Rheumatology [22], and age above 18 years old. SSc was classified as limited cutaneous (lcSSc) or diffuse cutaneous SSc (dcSSc) according to the criteria of LeRoy *et al*. [23]. Exclusion criteria were cigarette smoking, diabetes mellitus, hypercholesterolemia, or any associated severe disease (cancer, cardiac and pulmonary failure, myocardial infarction, angina pectoris). Furthermore, patients receiving statins, nitrates or non-steroidal anti-inflammatory drugs were excluded. All patients were asked to discontinue any vasodilator therapy given for Raynaud's phenomenon one week before inclusion and until the end of the study. Patients unable to discontinue vasodilator therapies during the study period were not included.

The onset of the disease was defined as the first occurrence of symptoms of SSc except for RP. Digital pitting scars, esophageal dysfunction and RP were diagnosed clinically. Skin thickness was quantified using the modified Rodnan skin score [24]. The diagnosis of pulmonary fibrosis was suspected on the basis of clinical data and systematic radiographs, and confirmed in all cases by computed tomography scans.

Primary RP was diagnosed according to the criteria of LeRoy and Medsger [25], including a normal nailfold capillaroscopy, the lack of antinuclear antibodies, no digital pitting scar and the lack of clinical symptoms of connective tissue disease.

This was a descriptive monocentric controlled study. All subjects gave informed written consent. The study was approved by the institutional review board of Grenoble University Hospital, France, on January 2004. Eligibility criteria and clinical status were assessed, and instructions for vasodilator therapy withdrawal were given to each subject. Twenty patients suffering from SSc were recruited from the Vascular Medicine Department. When a patient with SSc was enrolled, they were matched (sex and age ± 5 years) with a patient with Raynaud's phenomenon and a healthy volunteer. All patients with Raynaud's phenomenon and healthy volunteers were recruited through local newspaper advertisements.

All subjects arrived at the Clinical Research Center in Grenoble University Hospital between 8 a.m. and 9 a.m. in a fasting state, where the following measurements were performed within one day in a quiet room with a stable ambient temperature. After clinical examination, subjects were placed in a supine position with both forearms resting at heart level. Blood pressure and heart rate were determined and electrocardiography was performed, followed by laser Doppler measurements on the left arm. Venous blood samples were taken at fast for blood lipids and plasma glucose determination either before or after the laser Doppler measurements. Thereafter, subjects underwent echocardiography. Patients with SSc underwent pulmonary function testing.

#### Second study

Given the altered response to local heating we observed in SSc, we also tested in a pilot study whether this altered response was specific to this connective tissue disease. We studied 10 consecutive subjects with rheumatoid arthritis (RA) in the Rheumatology Department (Grenoble University Hospital, Grenoble, France) and 10 sex and age-matched (± 5 years) patients with primary Raynaud's phenomenon in the Inserm Clinical Research Center (Grenoble University Hospital, Grenoble, France), from April 2005 to May 2005. All subjects gave informed written consent. The study was approved by the institutional review board of Grenoble University Hospital, France, on April 2005. The criteria for inclusion in the study in the RA group were diagnosis of RA according to the American College of Rheumatology [26], and age above 18 years old. The criteria for inclusion in the study in the primary RP group were the same as in the main study. All subjects underwent thermal hyperhemia testing using the methodology detailed below.

### Laser Doppler measurements

Cutaneous blood flow was measured using a laser Doppler flowmeter (PeriFlux System 5000, Perimed, Järfälla, Sweden). A laser probe (PR457) was attached to the distal pad of the third left finger and left in place during all the laser Doppler measurements. Data from the laser Doppler flowmeter were interfaced to a personal computer through a converter using Perisoft^® ^data acquisition software.

Laser Doppler blood flow was recorded in mV, which are directly related to blood flow in the microcirculation of the surface tissue. Red blood cell flux values were divided by mean arterial pressure to yield a value of cutaneous vascular conductance expressed as mV/mm Hg. The expression of data in this manner takes into account any changes in blood flow due to change in blood pressure and also better reflects absolute changes in skin blood flow.

Following 30 minutes of rest, the hyperhemia was studied in the following sequence: post-occlusive hyperhemia with a 20 minute recovery period; sublingual nitroglycerin challenge with a 30 minute recovery period; and thermal hyperhemia. The recovery periods, determined in pilot experiments, were such that the cutaneous conductance returned to baseline values within these periods.

#### Post-occlusive hyperhemia

After 10 minutes of rest, to allow for the measurement of baseline cutaneous conductance, digital blood flow was occluded for 5 minutes by inflating a cuff placed on the left arm to 50 mm Hg above the systolic blood pressure. The cuff was then released and the flow responses were recorded. The amplitude of the response was determined by the peak hyperhemic conductance, expressed as an absolute value (mV/mm Hg). The kinetics of the response were determined by the time to peak hyperhemia and duration of hyperhemia, expressed in seconds.

#### Endothelium-independent response

Endothelium-independent vasodilation was tested 20 minutes later, following blood pressure and heart rate measurements. A single high dose of sublingual nitroglycerin (0.4 mg) was given. Digital skin blood flow was continuously recorded. The maximal effect was measured as the mean signal over a 1 minute period 4 minutes after nitroglycerin administration, similar to what is used for vasodilation of the brachial artery [27]. The amplitude of the response was determined by the 4 minute peak conductance, expressed as mV/mm Hg.

#### Thermal response

We measured microvascular response to local heating 30 minutes later. The PR457 laser probe was heated to 42°C for 30 minutes and then to 44°C for 5 minutes. Laser Doppler flow measured over the first 30 minutes is characterized in healthy controls by an initial peak within the first 10 minutes followed by a nadir and a final rise to a second peak that continues as a sustained plateau. Maximal skin blood flow is achieved by heating to 44°C. The amplitude of the response was determined by the 10 minute thermal peak, 10–30 minute thermal peak, and 44°C thermal peak conductances, expressed as absolute values (mV/mm Hg). The maximal effects were measured as the mean signal over a 1 minute period for the 10 minute thermal peak, and as the mean signals over a 3 minute period for the 30 minute and 44°C thermal peaks. In subjects without a clear-cut initial peak, the maximal value of the first 10 minutes was measured as the mean signal over a 1 minute period, corresponding to the highest mean within the 10 minute window. The kinetics of the response were determined by the time to first thermal peak, and the time to second peak when available. The time to first peak was determined from the onset of the probe heating to the first peak, or to the maximal value when no clear plateau was observed.

#### Reproducibility of laser Doppler measurements

Reproducibility was tested on 20 healthy subjects for the thermal hyperhemic response, and on 10 healthy subjects for the post-occlusive hyperhemic response. Post-occlusive hyperhemia and thermal hyperhemia were performed as detailed above. Each examination was repeated 1 day after the end of the first series on the same subject. For thermal hyperhemia, the median absolute difference for the time to first thermal peak was 13 s (10^th^-90^th ^percentile: 4–60) for a median of the means of 152 s (105–233). The median absolute difference for the 10 minute thermal peak was 4.5 mV/mm Hg (0.3–46) for a median of the means of 59 (20–81). For the post-occlusive response, the median absolute difference for the time to peak hyperhemia was 20 s (5–40) for a median of the means of 44 s (23–74). The median absolute difference for the peak hyperhemic conductance was 2 mV/mm Hg (0.5–9) for a median of the means of 46 (26–59). The coefficient of correlation for the time to first thermal peak and the 10 minute thermal peak was 0.89 and 0.65, respectively. The coefficient of correlation for the time to peak hyperhemia and peak hyperhemic conductance was 0.56 and 0.94, respectively. As correlation coefficients are poor indicators of reproducibility, Bland and Altman plots were constructed to measure the agreement between both measures. For the four measures, more than 95% of the differences were less than two standard deviations, and neither proportional error nor systematic errors were detected.

### Data analysis

The mean time to first thermal peak was 154 s (standard deviation 56) in the 20 healthy controls involved in the repeatability study. Sample size calculations were based on the main objective, that is, to detect a difference in the time to first thermal peak of at least 60 s between groups, with α = 0.05 and power (1-β) = 0.9.

Quantitative data were analyzed with the following nonparametric statistical methods: Kruskal-Wallis analysis of variance; Mann-Whitney test for between groups comparisons; Wilcoxon test for paired analysis; and Spearman rank correlation test for the relationship between quantitative variables. Proportions were compared by using Chi^2 ^tests or Ficher's exact test when appropriate. P-values less than 0.05, corrected by Bonferroni's method for multiple comparison, were considered significant. All quantitative data are expressed as the median, 10^th ^and 90^th ^percentiles. Qualitative data are expressed as number and percentage.

## Results

### Clinical and biological characteristics

The demographic, clinical and biological characteristics of the patients enrolled in the SSc study are listed in Table 1. Among patients with SSc, one patient was treated with methotrexate, one with cyclophosphamide and one with a synthetic antimalarial drug. One patient in the RP group and eight in the SSc group were on calcium channel blockers at the time of inclusion and one of each group was on buflomedil. Both calcium channel blockers and buflomedil were stopped 7 days before enrollment in the study. Arterial pulmonary hypertension was suspected in one SSc patient at the time of inclusion and confirmed by right heart catheterization.

In the second study, 10 patients with RA and 10 patients with primary RP were enrolled. Their clinical and biological characteristics are listed in Table 2. Among the patients with RA, all were receiving infliximab, 50% oral corticosteroids, 40% methotrexate and 30% non-steroidal anti-inflammatory drugs.

### Comparison of thermal hyperhemia in systemic sclerosis, primary Raynaud's phenomenon and healthy controls

**Figure 1:**
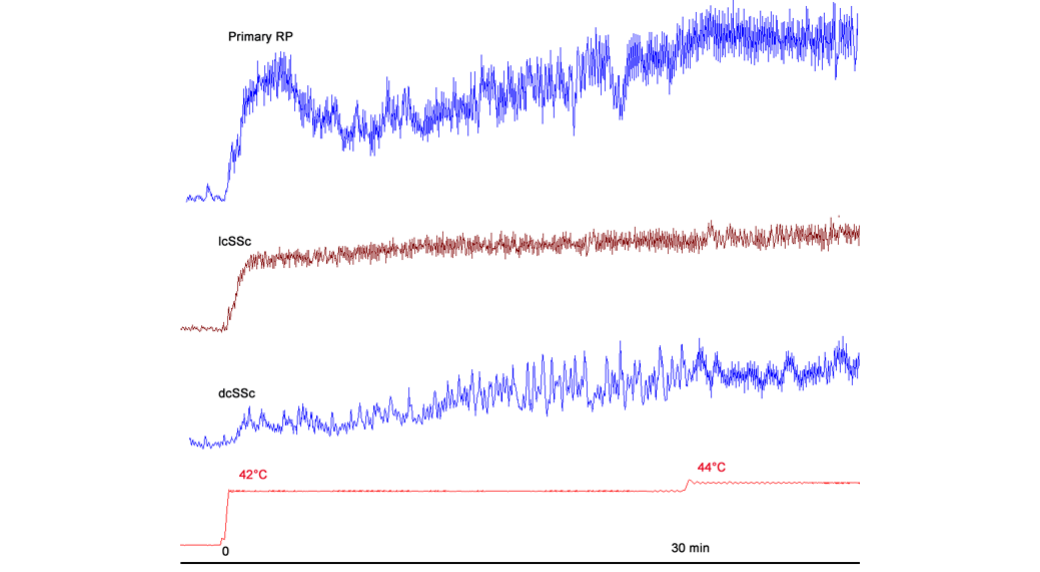
Thermal hypermemia in Raynaud's phenomenon (RP), limited and diffuse cutaneous systemic sclerosis (lcSSc and dcSSc). The patient with primary Raynaud's phenomenon has a normal response, including a first peak within the first 10 minutes and a nadir followed by a second plateau. This response is altered in the lcSSc patient, in which neither the initial cutaneous vascular conductance peak nor nadir is observed. The dcSSc patient shows a destructured response, including a delayed maximal response.

**Figure 2:**
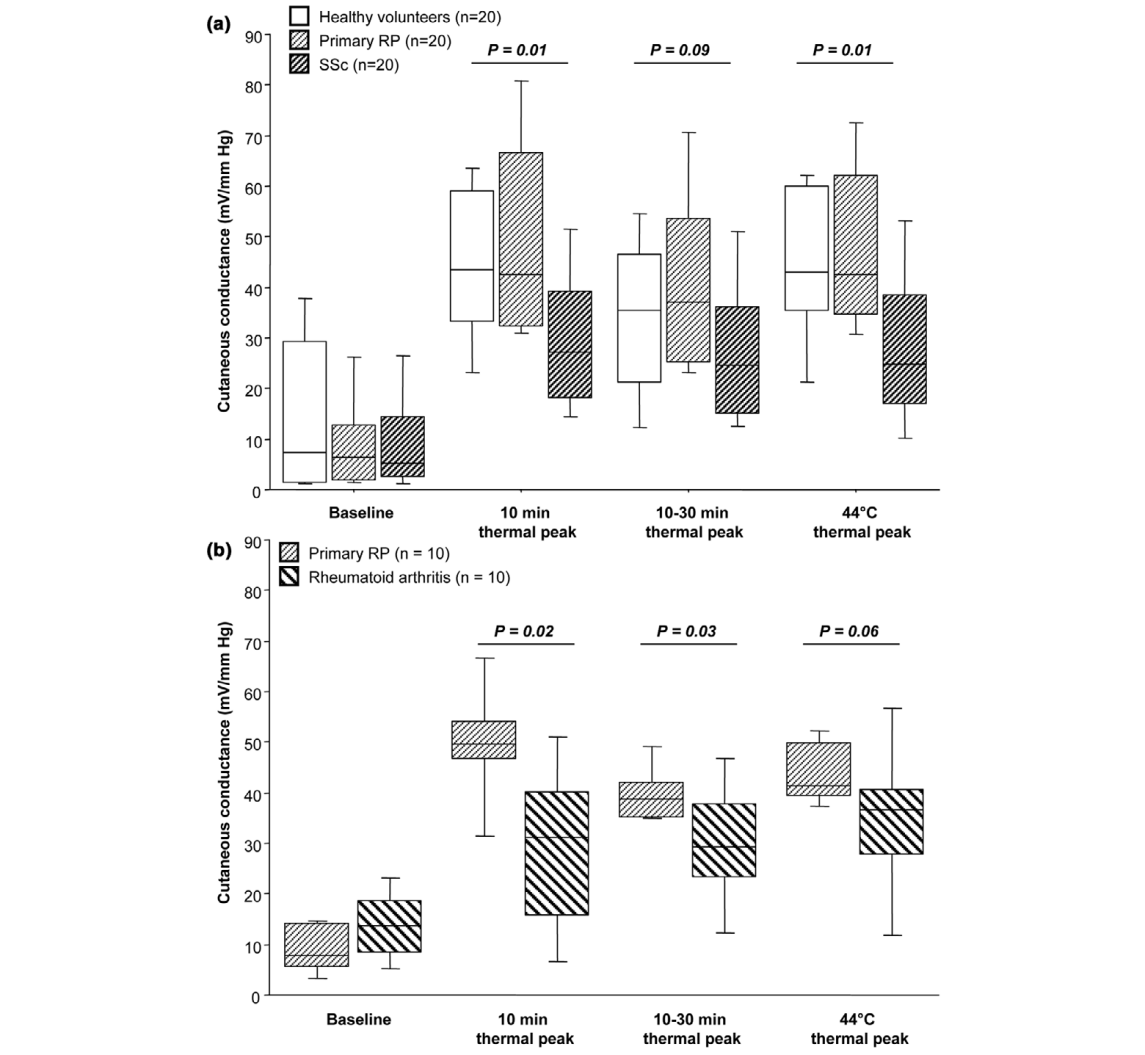
Amplitude of the thermal hyperhemia in systemic sclerosis (SSc) and rheumatoid arthritis. **(a) **Patients with systemic sclerosis (SSc), primary Raynaud's phenomenon (RP) and healthy controls. **(b) **Patients with rheumatoid arthritis (RA) and primary RP. Data are expressed as box plots in which 50% of values lie within the box. The horizontal line depicts the median value and the whiskers indicate the 10^th ^and the 90^th ^percentiles of all values.

Thermal hyperhemia was dramatically altered in terms of kinetics in patients with SSc (Table 3, Fig. 1). While 19 healthy volunteers and 18 patients with primary RP exhibited the classic response, including an initial peak within the first 10 minutes followed by a nadir and a second peak, this occurred only in four of the SSc patients (p < 0.0001). Similarly, a first peak occurred within 10 minutes in all healthy volunteers and all patients with primary RP, whereas an initial peak within 10 minutes was present in only 11 SSc patients (p < 0.0001). The absence of the first peak had a sensitivity of 80% and a specificity of 90% for differentiating SSc from primary RP, with an accurary of 85%, a positive likehood ratio of 8 and a negative likehood ratio of 0.22. Thermal hyperhemia was also altered in terms of amplitude (Table 3, Fig. 2).

The response to sublingual nitroglycerin did not significantly differ between groups (Table 3). The amplitude and kinetics of cutaneous vascular conductance response to occlusive hyperhemia was altered in patients with SSc (Table 3).

### Secondary objectives

The time to first thermal peak was correlated to the 10 minute thermal peak amplitude (r = 0.38; p = 0.003). It was also correlated to the time to the post-occlusive peak hyperhemia (r = 0.29; p = 0.03). When introduced in a linear regression analysis model, both the 10 minute thermal peak amplitude and the time to post-occlusive peak hyperhemia remained significant. No correlation was found, however, between the time to first thermal peak and the post-occlusive peak hyperhemic conductance.

In the SSc group, the time to the first thermal peak was correlated to the Rodnan's modified skin score (r = 0.6; p = 0.005). The kinetics of both the thermal challenge and post-occlusive hyperhemia were altered in patients with dcSSc in comparison with lcSSc: the time to first thermal peak was 255 s (162–986) and 1395 s (412–1780) in patients with lcSSc and dcSSc, respectively (p = 0.003), while the time to post-occlusive peak hyperhemia was 78 s (13–250) and 159 s (123–335), respectively (p = 0.05). In contrast, the amplitudes in both tests were similar in patients with lcSSc and dcSSc.

### *Post hoc *analysis

To test whether the lower conductance in response to heat in the SSc group was just a consequence of lower microvascular density, we normalized the thermal hyperhemia to the endothelium-independent nitroglycerin response. The ratio of the 44°C thermal peak minus baseline to the nitroglycerin peak minus baseline was 14 (3–131), 19 (3–225) and 1.8 (0–20) in healthy subjects, the RP group, and the SSc group, respectively (p < 0.05).

We tested as a *post hoc *analysis whether the maximal conductance between the whole thermal challenge (0 to 30 minutes) would differ between groups. The maximal conductance was 43.4 (28–63), 42.6 (31–81) and 27.1 (17.6–52.3) mV/mm Hg in the healthy volunteers, primary RP group and the SSc group, respectively (p = 0.001).

### Receiver-operating characteristic curve analyses

Receiver-operating characteristic curves for the diagnosis of SSc in subjects with RP were plotted for the primary RP and SSc patients. The area under the curve for the time to first thermal peak was 0.87 (95% confidence interval 0.71–0.95), and for the 10 minute thermal peak was 0.79 (95% confidence interval 0.61–0.89). The area under the curve for the time to peak hyperhemia was 0.75 (95% confidence interval 0.58–0.87) and for the peak hyperhemic conductance was 0.47 (95% confidence interval 0.31–0.64). A time to first thermal peak value higher than 180 s had a sensitivity of 90% and a specificity of 70% for differentiating SSc from primary RP, with an accurary of 80%, a positive likehood ratio of 3 and a negative likehood ratio of 0.14.

### Specificity of the thermal response

To test whether other connective tissue disorders may share the same pattern of altered response, we conducted a second study in which thermal hyperhemia was performed in 10 patients with RA and 10 patients with primary RP. The thermal hyperhemia was altered in terms of amplitude in patients with RA (Fig. 2). Conversely, the thermal hyperhemia was not altered in terms of kinetics in patients with RA. The time to thermal peak was 222 s (116–1684) in RA versus 200 s (134–266) in primary RP (not significantly different). Whereas all patients with primary RP exhibited the classic response, including an initial peak within the first 10 minutes followed by a nadir and a second peak, this occurred in only 7 of the 10 RA patients (p = 0.06).

## Discussion

Our study shows that hyperhemia to local heating is dramatically altered in terms of kinetics and amplitude in patients with SSc in comparison with patients with primary RP, the latter of which behave similarly to healthy controls. Furthermore, while the kinetics of the thermal response distinguish patients with SSc from among those presenting with a RP, the altered pattern of response is shared with other connective tissue diseases such as RA.

Scleroderma spectrum disorders are heterogeneous diseases with a large variation of clinical manifestations in individual patients. At one end of the spectrum is undifferentiated connective tissue disease, which includes the presence of RP and a typical capillaroscopy scleroderma pattern or positivity for Scl-70 or anticentromere antibodies; at the other end of the spectrum is SSc. In this study, we chose to include patients fulfilling the American College of Rheumatology criteria with no evidence of overlap syndrome, in order to study definite SSc cases rather than overlap or undifferentiated connective tissue disease. Although laser Doppler has been widely used to assess microvascular function in SSc, its utility in a clinical setting has remained poor to date. Most groups have used cold challenges, for which no specific pattern of response has been observed. For example, in a previous study using laser Doppler perfusion, we showed that although patients with SSc had a lower cutaneous blood flow at baseline, the variation of response was similar to subjects with primary RP and healthy controls when exposed to whole body cooling [13]. Conversely, post-occlusive and thermal hyperhemia provide information on the endothelial and microvascular function. Until recently, the mechanisms mediating post-occlusive hyperemia were still unknown. Two recent studies, however, specifically tested the contribution of two major endothelium-derived mediators, specifically NO and prostacyclin. Cyclooxygenase inhibition decreases the post-occlusive hyperemic response [16], whereas NO synthase inhibition does not affect it [16,18]. Furthermore, NO concentration does not increase during post-occlusive hyperhemia [28]. These studies clearly show that the endothelial part of the response is prostacyclin but not NO dependent. Conversely, the sustained plateau phase in response to local hyperthermia is NO-dependent [19,20]. Therefore, these tests clearly do not explore the same pathways of endothelial function. Although we show that the kinetics of the response in both tests are correlated, this correlation is weak.

Although post-occlusive hyperhemia is altered in patients with SSc [12,14], the response is also weakly altered in subjects with primary RP [14], something that we also observed in the present study. In contrast, we show that thermal hyperhemia is normal in subjects with primary RP. As both functional tests explore different mediators of endothelial function, this suggests that thermal hyperhemia could be more specific for SSc or other connective tissue disease vascular dysfunction, whereas post-occlusive hyperemia could be more sensitive to the RP itself. Our descriptive data, however, do not elucidate the specific mechanisms of the altered thermal hyperhemic response. Indeed, most of the patients with SSc do not exhibit the classic initial peak followed by a nadir. Whereas the impaired thermal hyperhemia in terms of amplitude could be related to a decreased ability to release NO, the altered kinetics of response could be related either to a local impaired axon reflex mediated vasodilation or to an inability to increase cutaneous blood flow due to macrovascular disease, including ulnar and digital artery narrowing. Further studies are also required to determine whether this impairment is correlated to the morphological changes or whether it precedes them.

A concern about laser Doppler flowmetry is reproducibility. We found the reproducibility to be correct for the first thermal peak, the 10 minute thermal peak and the post-occlusive peak hyperhemic conductance but not for the post-occlusive time to peak hyperhemia. A significant limitation when using laser Doppler flowmetry is the normalization to baseline. When using normalization, the ratio is highly dependent on the baseline values, which are very variable, leading to a poor reproducibility. In previous experiments using microdialysis, we normalized to a maximum dilation observed during sodium nitroprusside infusion [18], but whereas this is the ideal way to normalize the response, this can not be done in a clinical setting given the invasive approach. In this study we instead used an indirect approach (a sublingual spray of nitroglycerin) that induces an endothelium independent vasodilation. The effect of nitroglycerin was similar in the three groups studied and was also similar to what was observed after brachial artery infusion of sodium nitroprusside [3]. As the ratio of thermal hyperhemia over the nitroglycerin hyperhemia was much lower in the SSc group compared to the other groups, we may conclude that the lower maximal amplitude of thermal hyperhemia is not only related to the decreased capillary density, but to a decreased response to the thermal challenge.

To test whether an altered response to thermal hyperhemia was specific for SSc, we carried out a second study in which patients with RA suffering from Raynaud's phenomenon were enrolled. A potential pitfall in this study was the inclusion of patients receiving infliximab, which may itself improve endothelial function [29]. Furthermore, patients had a minimally active disease as suggested by their median disease activity score of 28, and the results may differ in patients with active RA. We clearly showed, however, that thermal hyperhemia is altered in RA as well as in SSc in terms of amplitude. As a consequence, an altered vascular response to local heating seems to be a hallmark of secondary RP, and is not disease specific. Indeed, there is strong evidence that endothelial dysfunction is present in patients with RA with both high and low disease activity scores [29,30]. While these two studies were performed using brachial artery flow mediated dilation, that is, analysis of a large conductance artery, we show that the NO-dependent second peak to local heating is impaired in RA, suggesting an abnormality of the microvascular endothelium. Conversely to the altered response in terms of amplitude, we were not able to demonstrate a difference in terms of kinetics in RA in comparison with primary RP. As the number of patients studied was small, further studies are required to determine whether this is due to a lack of power to detect a weak difference, or to a real similarity in the kinetic response. We still need to determine in future studies whether the abnormal response to local heating in SSc and RA differs with respect to the pathogenic mechanisms.

Noninvasive determination of microvascular dysfunction as an early event in the disease process remains a great challenge. Indeed, most patients with SSc initially only present an RP, which preceeds the cutaneous and/or pulmonary fibrosis. To date, nailfold capillaroscopy and detection of autoantibodies are used to determine which patients are susceptible for the development of SSc. At this time, there is still place for a noninvasive functional test. Our data suggest that the response to local heating could help to distinguish patients with secondary RP among those presenting with RP. This has to be confirmed in a prospective follow-up cohort study, however, to determine whether the thermal test could prospectively discriminate those patients presenting with RP who will develop clinical signs of connective tissue diseases. We are also planning a study to include patients with RP presenting with nailfold capillaroscopy abnormalities and/or autoantibodies that do not meet the criteria for SSc diagnosis to determine whether the alteration of the thermal response is an early event in the disease course.

## Conclusion

An effective test for endothelial and microvascular function would be an important advance in the diagnosis and monitoring response to treatment in scleroderma spectrum disorders. Thermal hyperhemia is dramatically altered in patients with secondary RP in comparison with subjects with primary RP. Further studies are required to determine the mechanisms of this altered response, and whether it may provide additional information in a clinical setting.

## Abbreviations

dcSSc = diffuse cutaneous systemic sclerosis; lcSSc = limited cutaneous systemic sclerosis; NO = nitric oxide; RA = rheumatoid arthritis; RP = Raynaud's phenomenon; SSc = systemic sclerosis.

## Competing interests

The authors declare that they have no competing interests.

## Authors' contributions

AB recorded measurements for all subjects enrolled in the first study, performed their laser Doppler flowmetry, and drafted the manuscript. MS recorded measurements for all subjects enrolled in the second study, performed their laser Doppler flowmetry, and drafted the manuscript. PC and CM discussed the study design, the methods used, and the results of the measurements. FSR discussed the study design of the first study and helped with patient enrolment. LG and CD discussed the study design of the second study and helped with patient enrolment. JLC developed the two studies, enrolled the patients in the first study, supervised the work of AB and MS and helped to draft the manuscript. All authors read and approved the final manuscript.

## Figures and Tables

**Table 1:** Demographic, clinical and biological characteristics of patients enrolled in the first study

	Healthy controls (n = 20)	Primary RP (n = 20)	SSc (n = 20)
Age (years)	47 (37–58)	46 (37–59)	50 (33–61)
Female	18 (90%)	18 (90%)	18 (90%)
Body mass index (kg/m^2^)	22 (20–29)	20 (18–23)	23 (20–26)
Raynaud's phenomenon	0	20 (100%)	20 (100%)
Median Raynaud's disease duration (years)	0	18 (6–43)	8 (3–22)
Raynaud's phenomenon: median number of fingers involved	0	8 (6–10)	10 (8–10)
Raynaud's phenomenon: thumb involved	0	7 (35%)	18 (90%)
Raynaud's phenomenon: feet involved	0	8 (40%)	16 (80%)
Median disease duration (years)	NA	NA	5 (0,5–16)
Digital pitting scars	0	0	8 (40%)
Sclerodactyly	0	0	20 (100%)
Median Rodnan modified skin score	0	0	6 (2–29)^a^
dcSSc/lcSSc	0/0	0/0	6/14
Pulmonary fibrosis	0	0	7 (35%)
Esophageal dysmotility	0	0	13 (65%)
Median creatinine clearance (ml/min)	81.7 (66–119)	74.9 (68–97)	87.9 (79–139)
Microalbuminuria (mg/l)	11 (11–17.6)	11 (11–20.8)	11 (11–17.7)
Median cardiac rate (beat/min)	62 (49–77)	69 (52–92)	65 (57–78)
Median systolic/diastolic blood pressure (mm Hg)	111 (99–133)/ 66 (54–78)	107 (91–122)/ 62 (52–79)	106 (89–141)/ 67 (54–85)
Median oxygen saturation (%)	99 (97–100)	100 (97–100)	100 (96–100)
Autoantibodies			
Anti-centromere	0	0	8 (40%)
Anti-topoisomerase I	0	0	5 (25%)
Median plasma LDL cholesterol (g/l)	1.04 (0.7–1.4)	1.04 (0.6–1.4)	0.9 (0.6–1.2)
Median plasma triglycerides (g/l)	0.7 (0.3–1)	0.5 (0.4–0.9)	0.7 (0.3–0.9)
Median plasma glycemia (mmol/l)	4.7 (3.6–5.3)	4.6 (4–5.7)	4.6 (4.3–5.2)

Quantitative data are medians, with 10^th ^and 90^th ^percentiles in parentheses. Qualitative data are expressed as numbers. ^a^The median Rodnan modified skin score was 4 (2–11) for the limited cutaneous systemic sclerosis (lcSSc) and 25 (12–43) for the diffuse cutaneous systemic sclerosis (dcSSc). NA, not applicable.

**Table 2:** Demographic, clinical and biological characteristics of patients enrolled in the second study

	Primary RP (n = 10)	RA (n = 10)
Age (years)	55 (42–64)	53 (39–68)
Female	7 (70%)	7 (70%)
Body mass index (kg/m^2^)	22 (19–23)	25 (20–30)
Raynaud's phenomenon	10 (100%)	10 (100%)
Median disease duration (years)	NA	9 (4–36)
Rheumatoid factor	0	9 (90%)
Disease Activity Score 28	NA	2.4 (2.3–4.9)

Quantitative data are medians, with 10^th ^and 90^th ^percentiles in parentheses. Qualitative data are expressed as numbers. NA, not applicable.

**Table 3:** Induced hyperhemia in patients with systemic sclerosis or primary Raynaud's phenomenon and healthy controls

	Healthy controls (n = 20)	Primary RP (n = 20)	SSc (n = 20)	P-value
Amplitude of the thermal response (median cutaneous vascular conductance (mV/mm Hg), 10^th^-90^th ^percentile)				
Baseline	7.2 (1.1–38)	6.4 (1.4–26.3)	5.2 (1.2–25.9)	NS
10 min thermal peak	43.4 (23.2–63)	42.6 (31–80.7)	27 (14.7–51.4)^a^	0.01
10–30 min thermal peak	35.5 (12.2–55)	37.2 (24.3–70.6)	24.7 (13.5–51)	0.09
44°C thermal peak	43.1 (21.3–62.1)	42.6 (31.6–74.3)	25.4 (15–52.4)^a^	0.01
Kinetics of the thermal response (median time, seconds)				
Time to first thermal peak	190 (125–230)	160 (91–311)	391 (171–1483)^a^	0.0001
Time to second peak	1458 (867–1949)	1394 (967–1991)	-	NS

Amplitude of the response to sublingual nitroglycerin (median vascular conductance (mV/mm Hg), 10^th^-90^th ^percentile)				
Baseline	14.1 (1.1–54.2)	4.6 (0.8–32)	11.5 (1.4–38)	NS
4 min peak	16.5 (1.6–55)	9.1 (1.2–34.5)	16.8 (3.6–37.7)	NS

Amplitude of the post-occlusive response (median cutaneous vascular conductance (mV/mm Hg), 10^th^-90^th ^percentile)				
Baseline conductance	13 (1.3–49)	4.8 (1.1–32)	7.7 (1.4–30)	NS
Peak hyperhemic conductance	36.8 (11–65)	28.5 (9.3–49)	25.1 (9.5–50.2)^b^	0.05
Kinetics of the post-occlusive response (median time (seconds) 10^th^-90^th ^percentile)				
Time to peak hyperhemia	34.3 (17.4–76.5)	44.4 (19.8–104)	108.5 (15–287)^a^	0.003
Duration of hyperhemia	165 (75–277)	150 (97–345)	225 (82–345)	NS

Data are medians, with 10^th ^and 90^th ^percentiles in parentheses. Cutaneous blood flow is expressed as cutaneous conductance (mV/mm Hg). Local heating at 42°C was applied for 30 minutes, followed by 5 minutes heating at 44°C. The cutaneous blood conductance was measured on the left middle fingerpad. ^a^P < 0.05 following Bonferroni's correction versus healthy controls and versus primary Raynaud's phenomenon (RP), Mann-Whitney test. ^b^P < 0.05 following Bonferroni's correction versus healthy controls. SSc, systemic sclerosis.
